# Synergistic STING activation and oxidative cascades-induced ferroptosis drive tumor microenvironment remodeling by engineered manganese nanoreactors

**DOI:** 10.1016/j.redox.2025.103977

**Published:** 2025-12-15

**Authors:** Wa Li, Zihui Tang, Jiyang Xue

**Affiliations:** a Department of Medical Affairs, Shanghai First Maternity and Infant Hospital, School of Medicine, Tongji University, Shanghai, 200092, PR China; b Department of Pharmacy, Shanghai First Maternity and Infant Hospital, School of Medicine, Tongji University, Shanghai, 200092, PR China; c Department of Stomatology, Shanghai East Hospital, School of Medicine, Tongji University, Shanghai, 200120, PR China; d Department of Medical Oncology, Cancer Center, Shanghai General Hospital, Shanghai Jiao Tong University School of Medicine, Shanghai, 201620, PR China

**Keywords:** Chemodynamical therapy, Manganese nanoreactors, Immunogenic cell death, STING pathway, Ferroptosis

## Abstract

In head and neck squamous cell carcinoma, a “cold” (immune-desert) tumor microenvironment promotes immunosuppression, which is a critical driver of disease recurrence and therapeutic resistance. To address this challenge, we develop an innovative strategy to remodel the tumor immune microenvironment by disrupting intracellular redox balance to induce ferroptosis and immunogenic cell death, synergistically activating STING pathway to facilitating the transition of tumors from a “cold” to a “hot” immunophenotype. In this study, hyaluronic acid-functionalized hollow manganese dioxide nanoparticles loading β-lapachone (hMnL), engineered for targeted chemo-immunotherapy is constructed. *In vitro* investigations reveal that hMnL induces robust reactive oxygen species (ROS) generation, triggering ferroptosis and immunogenic cell death. Concurrently, Mn^2+^ ions released from hMnL in response to the acidic tumor microenvironment activate the STING pathway, fostering dendritic cell (DC) maturation and M1 macrophage polarization. Activation of the ferroptosis and immune-related pathways was indicated by transcriptome sequencing, which identified significantly differentially expressed genes (e.g., *Fth1*, *Hmox1*, *Calr*). *In vivo*, hMnL exhibits superior tumor-targeting efficacy and sustained intratumoral retention, culminating in potent tumor growth suppression. Furthermore, hMnL activates STING pathway in tumor, leading to enhanced CD8^+^ T cell infiltration, and a marked reduction in regulatory T cell (Treg) populations. Additionally, hMnL also shows good immunoprotective effects and long-term biosafety. These findings establish hMnL as a promising therapeutic platform that integrates targeted chemotherapy with immune modulation, offering a potent strategy to overcome immunosuppression and improve clinical outcomes in cancer.

## Graphical abstract

**Figure:**
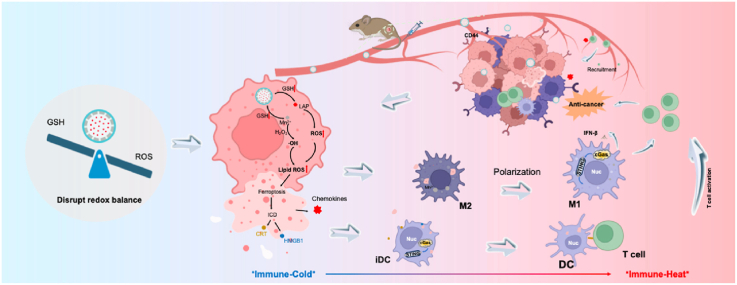

## 1 Introduction

Head and neck squamous cell carcinoma (HNSCC) represented a highly aggressive malignancy with pronounced invasive and metastatic potential [1]. Conventional therapeutic interventions, including radiotherapy, surgical treatment, and chemotherapy, while providing partial tumor control, were frequently compromised by therapeutic limitations such as locoregional recurrence, distant metastasis, and suboptimal therapeutic efficacy [2]. In light of these clinical challenges, the development of novel therapeutic strategies, has emerged as a pivotal research paradigm in contemporary oncological investigations.

Recently, immunotherapy had emerged as a transformative paradigm in cancer therapeutics, revolutionizing conventional cancer treatment modalities [3,4]. Nevertheless, the therapeutic efficacy of recent immunotherapeutic approaches in HNSCC, remained undesired, predominantly attributable to the intricate tumor immunosuppressive microenvironment and sophisticated immune evasion mechanisms [5,6]. There was a substantial infiltration of immunosuppressive cellular components within the TME, including an elevated prevalence of regulatory T cells (Tregs) and myeloid-derived suppressor cells (MDSCs) in HNSCC tissues [7]. These immunosuppressive populations exerted their inhibitory effects through the secretion of potent immunosuppressive cytokines, including transforming growth factor-beta (TGF-β) and interleukin-10 (IL-10), thereby creating an immunologically privileged niche for tumor progression [8,9]. Consequently, current research endeavors increasingly focused on developing innovative therapeutic strategies to reprogram the tumor immunosuppressive microenvironment and enhance antitumor immunity [[10], [11], [12]]. These approaches included combination therapies targeting multiple immune pathways and development of novel immune modulators to overcome therapeutic resistance and improve clinical outcomes in this challenging malignancy [13,14].

STING (Stimulator of Interferon Genes) pathway represented a pivotal mechanism in innate immune surveillance and had emerged as a transformative target in contemporary tumor immunotherapeutic strategies [[15], [16], [17], [18], [19]]. Functioning as a cytosolic DNA sensor, this pathway orchestrated robust downstream immune activation through the detection of genomic instability and aberrant nucleic acid accumulation, thereby initiating a cascade of antitumor immune responses [20]. Substantial preclinical evidence had demonstrated that pharmacological activation of the STING signaling axis could significantly enhance tumor immunogenicity, potentiate cytotoxic T lymphocyte infiltration, and suppress both primary tumor progression and metastatic dissemination [[21], [22], [23]]. These immunomodulatory effects positioned the STING pathway as a promising therapeutic target for reprogramming the tumor immunosuppressive microenvironment and eliciting durable antitumor immunity. However, current small-molecule STING agonists were hampered by suboptimal pharmacokinetic profiles, including rapid enzymatic degradation, systemic clearance, and limited bioavailability, resulting in insufficient intratumoral drug accumulation [24,25]. Innovative approaches, for example, nanoparticle-based delivery platforms were currently under intensive investigation to overcome these pharmacological barriers and maximize the therapeutic potential of the STING pathway modulation.

Nanotechnology had revolutionized oncological therapeutics by addressing limitations of conventional immunotherapy [26]. Engineered nanocarriers enabled targeted drug delivery to tumors, improving bioavailability and reducing toxicity [[27], [28], [29], [30]]. Manganese-based nanoparticles, in particular, had gained attention for their dual role in drug delivery and immunotherapy [31]. These nanoparticles exhibited unique catalytic properties that could remodel the tumor immunosuppressive microenvironment, while simultaneously enhancing immune cell activation through STING pathway modulation [32]. The intrinsic biological significance of Mn^2+^ ions, as essential cofactors in cellular signaling pathways, further amplified their therapeutic potential. Mechanistically, Mn^2+^ ions functioned as critical allosteric regulators of cyclic GMP-AMP synthase (cGAS), enhancing its affinity for double-stranded DNA (dsDNA) and catalytic efficiency in cyclic GMP-AMP (cGAMP) synthesis [33]. This molecular interaction significantly amplified the downstream STING signaling cascade, resulting in robust type I interferon production and antitumor immunity. Manganese nanoparticles uniquely combined targeted delivery with STING pathway activation, offering a breakthrough in cancer immunotherapy. This dual functionality addresses conventional STING agonist limitations and advances precision immunotherapy, potentially overcoming current therapeutic barriers. Thus, manganese-based nanoplatforms were set to transform tumor immunotherapy with their multifunctional and precise immunomodulatory effects.

The complexity of tumor immune responses challenged durable outcomes from monotherapeutic strategies. Additionally, prolonged drug administration could lead to reduced sensitivity in tumor cells and even induce drug resistance, resulting in limited therapeutic efficacy. Therefore, devising targeted interventions tailored to the growth characteristics of tumors to induce death represented an effective strategy. Ferroptosis, a novel form of regulated cell death driven by lipid peroxidation that had been extensively studied, could effectively target tumor cells resistant to conventional therapies, providing a novel approach to overcome drug resistance and enhance therapeutic efficacy in cancer treatment. Tumor cells relied on elevated reactive oxygen species (ROS) for proliferation, making ROS modulation a promising approach [34]. β-Lapachone (LAP), an FDA-approved small molecule, targets NAD(P)H: quinone oxidoreductase-1 (NQO1), which overexpressed in cancer cells (5–200 × higher than in normal tissues) [35,36]. NQO1 reduced LAP to an unstable intermediate, generating ROS through redox cycling. This oxidative stress caused DNA damage and PARP1 hyperactivation, inducing programmed cell death. The tumor-selective action of LAP, enabled by differences in NQO1 expression, led to a favorable therapeutic index, which maybe related with lipid peroxidation to further induce ferroptosis.

**Fig. 1:**
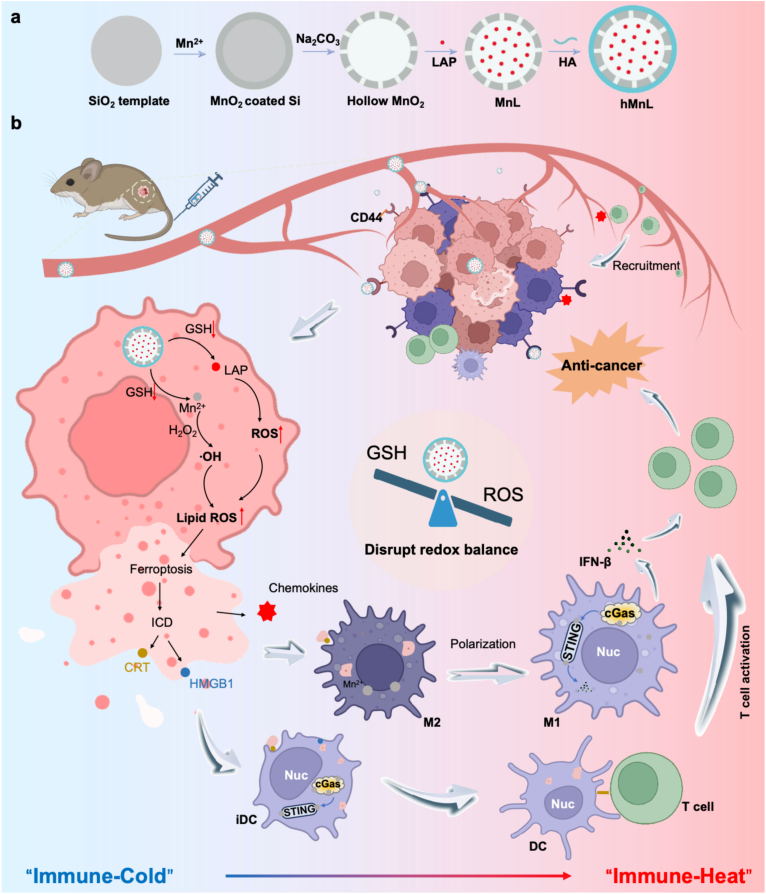
Schematic diagram of synthesis of hMnL and *in vivo* anti-tumor therapy. a) A manganese dioxide-based nanomedicine loaded with LAP was synthesized using a template method, designed to target cancer cells. b) Following intravenous injection, hMnL was specifically delivered to tumor tissues, mediated by HA. hMnL binded to CD44 molecules, which were highly expressed in tumor tissues, and subsequently internalized into tumor cells to exert its anti-tumor effects: Under the influence of GSH in the tumor, hMnL disintegrated to release LAP and Mn^2+^, which could perform CDT to induce tumor cell ferroptosis and ICD. Subsequent immune responses were triggered including induction of DC maturation and activation of T cells. Polarization of macrophages and activation of the STING pathway, enhancing the phagocytosis function of macrophages towards tumor cells. Secretion of IFN-β further enhanced the activation of T cells. Activated T cells exerted cytotoxic effects on tumor cells.

Based on the analysis above, the present investigation endeavored to engineer a sophisticated targeted drug delivery platform capable of achieving triple therapeutic objectives: 1) precise tumor-specific drug delivery, 2) induction of tumor cell ferroptosis and ICD by oxidative cascades, and 3) potent activation of the STING pathway, thereby augmenting immunotherapeutic efficacy in cancer. This approach was anticipated to improve the tumor immunosuppressive microenvironment, amplify tumor-specific immune responses, and ultimately increase clinical outcomes, while establishing a novel therapeutic paradigm for cancer immunotherapy. This engineered nanosystem—HA-functionalized manganese oxide nanoparticles encapsulating LAP, demonstrated remarkable TME-responsive characteristics, enabling spatiotemporally controlled release of both therapeutic payloads (Fig. 1a). The nanosystem induced cancer cell ferroptosis and ICD, while the liberated Mn^2+^ potentiate STING pathway activation, thereby orchestrating a synergistic chemo-immunotherapeutic effect (Fig. 1b). This innovative strategy represented a significant advancement in HNSCC immunotherapy, offering a promising therapeutic approach that integrated targeted chemotherapy with enhanced immune activation for improved clinical outcomes.

## 2 Materials and methods

### 2.1 Materials

Reactive oxygen species assay kit, Annexin V-FITC/PI, Calcein/PI and cell counting kit-8 (CCK-8) were purchased from Beyotime Biotechnology Co., Ltd. Manganese chloride, potassium permanganate, tetraethyl orthosilicate (TEOS), (3-Aminopropyl) triethoxysilane (APTES), hyaluronic acid, ethanol, sodium carbonate was provided by Shanghai Aladdin Biochemical Technology Co., Ltd. Fluorescently labeled antibodies were purchased from BioLegend, Inc. JC-1 Mitochondrial Membrane Potential Assay Kit and Cy7 was provided by MedChemExpress LLC. Mouse-reactive STING pathway antibody sample kit was purchased from Cell Signaling Technology. Triton × 100, CXCL10, IFN-γ, IL-12, TNF-α, IL-6, IL-10 and IFN-β Elisa kit was purchased from Beijing Solarbio Science & Technology Co., Ltd.

### 2.2 Synthesis of hMn, MnL and hMnL

5 mL of Triton X-100, 25 mL of hexane, and 5 mL of n-butanol were mixed thoroughly in a flask, followed by the immediate addition of 7 mL of ammonia solution. The mixture was stirred for 2 h, and then 5 mL of TEOS and 1 mL of APTES were added. After reacting for 24 h, the mixture was centrifuged at 12, 000 rpm for 10 min, and washed three times with ethanol to yield silica nanoparticles.

The obtained silica nanoparticles were then resuspended in water and a solution of potassium permanganate (10 mg/mL) was added dropwise. After reacting for 6 h, the mixture was centrifuged to collect the bottom particles. 2 M Na_2_CO_3_ was added and the solution was heated at 60 °C overnight. After centrifugation, the bottom particles were collected and washed 2–3 times with water, yielding hollow manganese dioxide nanoparticles. Afterwards, HA was added and mixed overnight to harvest HA coated hollow MnO_2_ nanoparticles (hMn). To synthesize MnL, 10 μL LAP (10 mg/mL) was added into hollow MnO_2_ nanoparticles and the mixture was stirred for 4 h. MnL was collected by centrifugation. Then, HA was added into the collected MnL to stir for overnight to harvest hMnL.

### 2.3 *In vitro* drug release

The hMnL was resuspended in PBS containing 10 mM GSH and loaded into a dialysis bag (3000 Da). The bag was then placed in 200 mL of PBS solution containing 10 mM GSH. At predetermined time intervals, 5 mL of the solution was collected and replaced with an equal volume of fresh solution. The drug content in the collected solution was measured, and a release curve was plotted. PBS without GSH was used as a control.

### 2.4 Stimuli-responsive disassembly

The hMn was resuspended in PBS, and GSH was added. The color change before and after the reaction was recorded using a camera. Simultaneously, the particle size before and after the reaction was measured using DLS. The post-reaction solution was collected, and morphological changes were observed by TEM.

### 2.5 Methylene blue (MB) assay

After hMn treated with GSH, the solution was centrifuged at 10, 000 rpm for 5 min, and the supernatant was collected. The supernatant was added to a solution containing sodium bicarbonate, and the pH was adjusted to 8. Subsequently, 150 μL of hydrogen peroxide (100 mM) and MB solution were added. The absorption peak of the solution was measured using a UV–visible spectrophotometer.

### 2.6 Surface CD44 detection

SCC-7 cells were collected and stained with CD44-APC antibody for surface labeling. After staining, the cells were washed with PBS to remove any unbound antibody. Subsequently, flow cytometry was used to analyze the expression level of CD44 on the cell surface.

### 2.7 Cell endocytosis assay

SCC-7 cells were seeded in culture dishes suitable for observation with CLSM. Once the cells reached approximately 90 % confluency, cells were preincubated with or without HA solution. After washing, fluorescently-labeled hMnL were added to the cells and incubated for 4 h. Following incubation, the cells were washed, fixed, and stained with DAPI to visualize the cell nuclei. The endocytosis process was observed by CLSM. Additionally, cells were collected and analyzed for endocytosis quantification using flow cytometry.

### 2.8 Intracellular ROS detection

SCC-7 cells were seeded in a 12-well plate and incubated overnight. After incubation, cells were exposed to different treatments for 12 h. The culture supernatant was discarded, and the cells were washed with PBS. Then, DCFH-DA probe solution was added, and the cells were incubated for 15–30 min to detect intracellular ROS levels. After washing with PBS, cells were collected and analyzed for ROS levels using flow cytometry. For further visualization of ROS production, cells were seeded in dishes and observed under CLSM to examine fluorescence intensity changes under various treatments.

### 2.9 Cytotoxicity, apoptosis/necrosis and live/dead analysis

SCC-7 cells were seeded in a 96-well plate, and once cells reached 90 % confluency, MnL and hMnL with different concentration gradients were added into cells. After 24 h of incubation, cell viability was assessed using the CCK-8 assay. To further investigate apoptosis and necrosis, cells were treated with hMn, LAP, hMnL. Afterwards, cells were washed with PBS and collected. Then, cells were then stained with Annexin V-FITC/PI to analyze for apoptosis and necrosis by flow cytometry. To explore cell death mechanisms, cells were also seeded in dishes, treated with Calcein-AM/PI, and observed under CLSM to assess live and dead cells.

### 2.10 Mitochondrial membrane potential assay

SCC-7 cells were planted in the dishes and after various treatments, cells were collected and resuspended. JC-1 dye (final concentration of 2 μM) was added, and the cells were incubated at 37 °C for 15–20 min. After incubation, cells were washed with PBS and analyzed for mitochondrial membrane potential changes using flow cytometry. To visualize changes in mitochondrial membrane potential more directly, cells were seeded in dishes with the same steps above and observed under the CLSM.

### 2.11 Determination of GSH and MDA levels

To further investigate the cell death mechanisms, cancer cells plated in multi-well plates were subjected to different treatments including PBS, hMn, LAP and hMnL. Subsequently, monochlorobimane (mBCI, final concentration 20 μM) was added and the cells were incubated for 30 min. After incubation, the cells were washed 2–3 times, followed by detection using flow cytometry. After the same treatments described above, cells were collected. The expression of GPX4 was then analyzed by WB, and the MDA content was determined according to the manufacturer's instructions of the assay kit.

### 2.12 *In vitro* BMDC/BMDM activation assay

The transwell system was used to evaluate the immunological effects of different treatments. SCC-7 cells were seeded in the upper chamber of the transwell system, and immune cells (BMDC or BMDM) were seeded in the lower chamber. After 12 h of incubation, hMn, LAP and hMnL were added to the upper chamber, and the incubation continued for a set period. Afterwards, the upper chamber was removed, and the lower chamber cells were further incubated for 24 h. The lower chamber cells were then collected, washed with PBS, and subjected to immunophenotyping: For BMDC, cells were first stained with Zombie Aqua fixable viability kit to distinguish live and dead cells, followed by staining with anti-CD11c-PE, anti-CD80-PerCP-Cy5.5, and anti-CD86-APC antibodies. All staining was performed at 4 °C for 30 min, and flow cytometry was used to analyze cell phenotype. For BMDM, cells were first stained with Zombie Aqua fixable viability kit to exclude dead cells, followed by staining with anti-CD11b-Pacific blue, anti-F4/80-FITC, anti-CD86-APC, and anti-CD206-PE antibodies, and analyzed by flow cytometry. Additionally, the upper chamber culture supernatant was collected for ELISA analysis of IFN-β, IL-6, and IL-10 cytokine secretion levels.

### 2.13 *In vivo* animal imaging experiment

For *in vivo* imaging, the hair on the back of C3H mice (6–7 weeks, female) was shaved, and a suspension containing five million SCC-7 cells was injected subcutaneously. Tumor volume was measured regularly using the formula: V=W × L^2^/2, where L was the longest diameter, and W was the shortest diameter. When the tumor volume reached around 100 mm^3^, Cy7-labeled hMnL or MnL nanoparticles were intravenously injected into the mice. Fluorescence images were captured at 2, 8, 12, 24, and 48 h post-injection. At 48 h, the mice were euthanized, and the tumor and major organs were harvested for further imaging and fluorescence intensity analysis. All animal experiments were performed under the Guide for the Care and Use of Laboratory Animals of the National Institutes of Health and approved by the Ethics Committee of Tongji University (Approval Number: TJBG13021101).

### 2.14 *In vivo* therapeutic experiment

In the tumor-bearing mouse model, treatments began when the tumor volume reached around 100 mm^3^. The mice were administrated with intravenous injections of PBS, hMn, LAP and hMnL, at Day 0 (the initiation of therapy). Subsequent treatments were performed every two days, and tumor size and mouse body weight were recorded. A total of five treatments were administered. On Day 24, the mice were euthanized, tumors were harvested to weigh. For further evaluation of the therapeutic effects, tumor tissue sections were subjected to H&E staining, TUNEL staining, and STING protein immunofluorescence. Simultaneously, tumor tissues were homogenized into suspensions, and the expression levels of proteins related to the cGAS-STING pathway were measured by Western blot and cytokine secretion (CXCL10, IFN-β, IFN-γ, IL-6, IL-12, TNF-α) in tumors were determined as well.

### 2.15 Immunological mechanism investigation

To investigate the immunological mechanisms of antitumor activity, tumor tissues were sectioned, CD8^+^ T cells within the tumor were labeled, observed by CLSM. Following that, tumors and lymph nodes were harvested and processed into single-cell suspensions using a 200 μm cell strainer. For DC staining in lymph nodes, cells were stained with anti-CD45-APC-Cy7, anti-CD11c-PE, anti-CD80-PerCp-Cy5.5, and anti-CD86-APC antibodies. For T cells in tumors, cells were stained with anti-CD45-APC-Cy7, anti-CD3-FITC, anti-CD4-PE, anti-CD8-APC, anti-CD25-Brilliant Violet 421™, and anti-Foxp3-APC antibodies. For macrophages in tumors, cells were stained with anti-CD45-APC-Cy7, anti-CD11b-Brilliant Violet 421™, anti-F4/80-FITC, anti-CD86-APC, and anti-CD206-PE antibodies. All staining was preceded by Zombie Aqua fixable viability kit to exclude dead cells. After staining, cells were washed with PBS, resuspended, and analyzed for immune cell phenotype using flow cytometry.

To investigate the immunoprotective effects of hMnL, tumor-bearing mice were monitored for 30 days post-treatment, followed by euthanasia. The spleens were then collected, and single-cell suspensions were prepared. The cells were stained according to the following protocol: anti-CD45-APC-Cy7, anti-CD3-FITC, anti-CD8-APC, anti-CD44-PE, anti-CD62L-APC. Afterwards, flow cytometry was used to analyze the subtype of immune cells.

### 2.16 Long-term biosafety evaluation

Healthy mice were intravenously administered with hMnL *via* the tail vein as described above. After four weeks, the mice weight was monitored and peripheral blood was collected for biochemical analysis. Concurrently, major organs (heart, liver, spleen, lung, and kidney) were harvested, sectioned, and stained with H&E for histopathological examination under a microscope.

## 3 Results and discussion

### 3.1 Preparation and characterization of engineering manganese nanoreactors

**Fig. 2:**
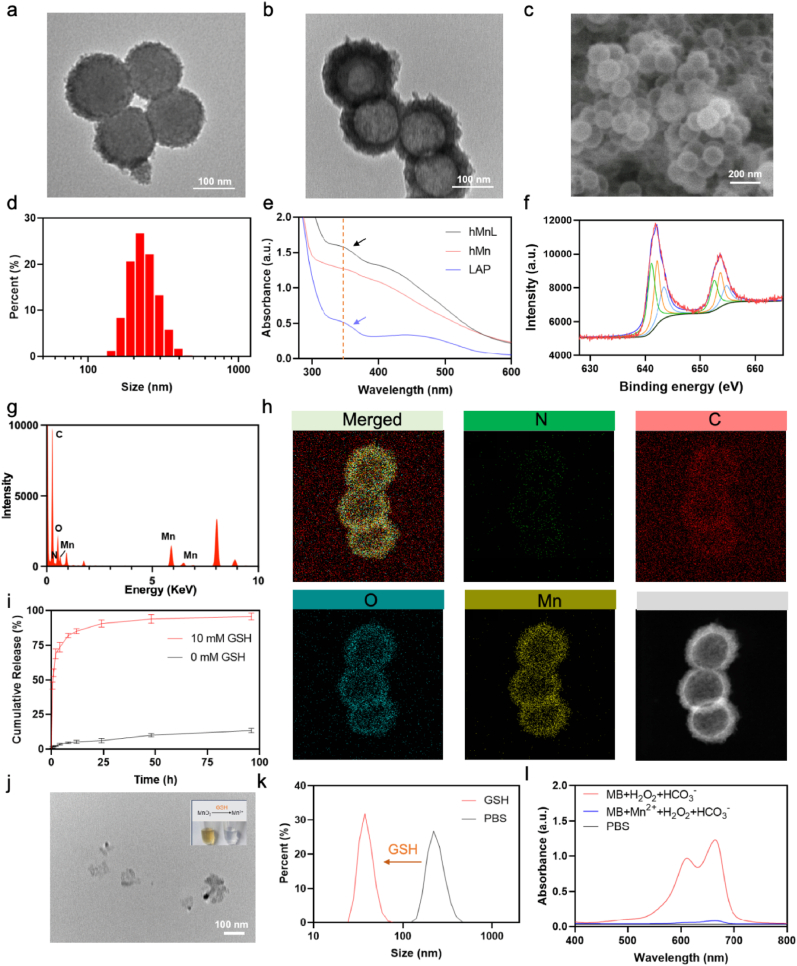
a) The morphology of the MnO_2_ coated SiO_2_ nanoparticles observed by TEM. b) TEM and c) SEM of HA coated hollow MnO_2_ nanoparticles. d) The hydrodynamic particle size of hMnL measured by DLS. e) UV absorption spectroscopy used to confirm the successful loading of LAP into the hMnL. f) X-ray photoelectron spectroscopy (XPS) employed to evaluate the valence state of Mn in hMnL. g) EDS and h) and the corresponding element mapping for the hMnL. i) The *in vitro* release curve of LAP from the hMnL under 0 or 10 mM GSH, with a detection period of 96 h. j) TEM of hMnL with the treatment of GSH and Mn element in the nanoparticles reduced to Mn^2+^ ions under GSH conditions, resulting in a color change in the solution (Inset). k) The size changes of hMnL monitored by DLS under GSH conditions. l) The released Mn^2+^ ions exhibited CDT effect by inducing the decolorization of MB solution, measured by UV spectrophotometer.

Manganese dioxide (MnO_2_) layer was fabricated on the surface of silica nanospheres by a template-assisted synthesis approach. The template was subsequently removed *via* treatment with sodium carbonate, resulting in the successful formation of hollow MnO_2_ nanoparticles. These hollow MnO_2_ nanoparticles were then loaded with the chemotherapeutic agent LAP and functionalized on their surface with HA to enable selective targeting of CD44. As depicted in Fig. 2a, MnO_2_ coated silica nanospheres were provided, whose morphology was observed by TEM. Fig. 2b was the morphology of HA coated hollow MnO_2_ nanoparticles. Through scanning electron microscopy (SEM) observations, hMnL exhibited a relatively uniform size distribution and a regular spherical morphology with smooth surfaces and well-defined edges (Fig. 2c). Dynamic light scattering (DLS) analysis revealed that the hMnL exhibited an average size of approximately 120 nm (Fig. 2d). Furthermore, UV spectroscopic measurements demonstrated a prominent absorption peak around 350 nm for both LAP and hMnL, thereby confirming the successful encapsulation of LAP within the nanoparticles (Fig. 2e). This formulation achieved a DLC of approximately 22.1 % and an EE of about 91.4 %. XPS spectra were recorded to determine the formation of MnO_2_. Two characteristic peaks attributed to Mn (IV) 2p_1/2_ and 2p_3/2_ can be found in the XPS spectra of hMnL (Fig. 2f). XPS results demonstrate the successful synthesis of MnO_2_. Fig. 2g presented the EDS analysis of hMnL. The spectrum revealed that hMnL primarily consisted of elements including C, N, O, S, and Mn. Furthermore, images of elemental mapping analysis confirmed the distribution of these elements on hMnL (Fig. 2h).

The manganese oxide within the nanoparticles was capable of degrading in the presence of high concentrations of glutathione (GSH) within the intracellular environment, a process that could be verified through *in vitro* release experiments (Fig. 2i). The results indicated that under the influence of 10 mM GSH, LAP was continuously and efficiently released, with a release rate approaching 100 % within 48 h. In contrast, under physiological conditions, the release rate of LAP remained minimal. Subsequently, the morphology of the nanoparticles treated with GSH was observed TEM. As shown in Fig. 2j, the nanoparticles underwent collapse and fragmentation, losing the original regular morphology. To more intuitively observe this behavior, the transformation of hMn from light yellow oxidized state to the clear divalent Mn^2+^ ion form under GSH treatment was visually evident (Fig. 2j, Inset). Additionally, a reduction in particle size after the treatment with GSH measured by DLS (Fig. 2k). These findings suggested that the nanoparticles could degrade into Mn^2+^ ions within tumor cells, facilitating the release of LAP to exert antitumor effects. To investigate the potential Fenton-like effect of the released Mn^2+^ ions and their ability to generate hydroxyl radicals, methylene blue (MB) dye was employed as a reaction monitor. As shown in Fig. 2l, the Mn^2+^ ions released upon nanoparticle degradation within tumor cells significantly caused MB decolorization, exhibiting a strong chemodynamic therapy (CDT) effect, which was conducive to the CDT of tumors *in vivo*. To evaluate the stability of hMnL, DLS was employed to monitor the hydrodynamic size and polydispersity index (PDI). The results showed no significant changes in the size or PDI values of the nanoparticles, indicating the excellent stability (Fig. S1). The variations in size and PDI remained within the margin of error, with no observable aggregation or precipitation. These findings demonstrated that hMnL possessed remarkable stability, making them suitable for further application studies.

### 3.2 Cell uptake and cytotoxicity of hMnL

**Fig. 3:**
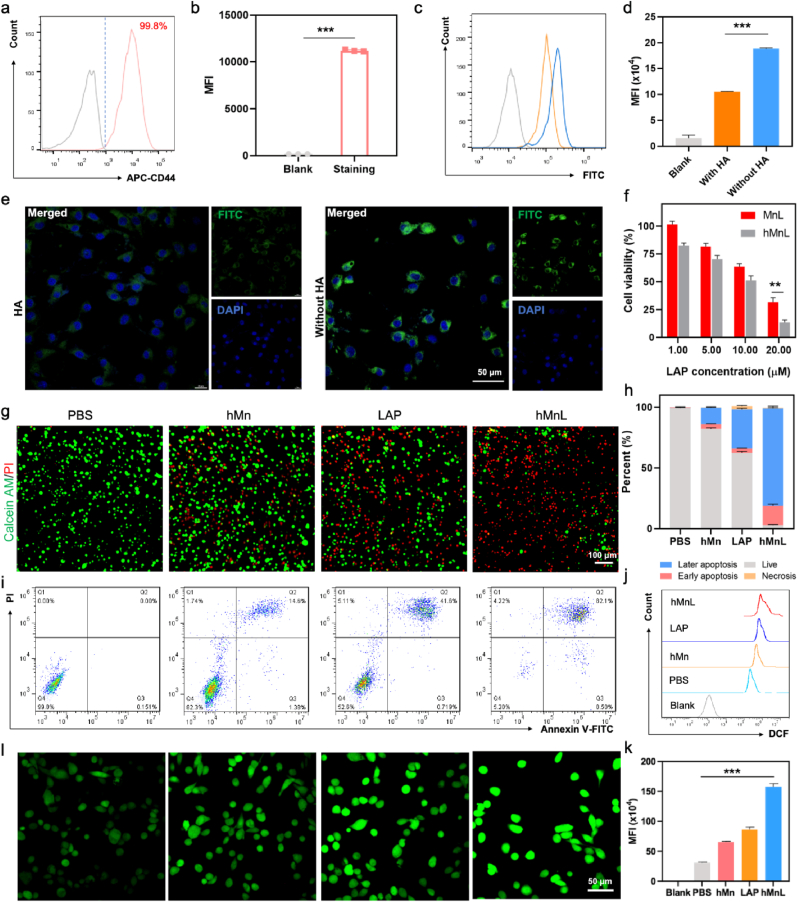
a) The expression level of CD44 in SCC-7 cells assessed by flow cytometry and b) the statistical graph of mean fluorescence intensity. Fluorescence-labeled hMnL incubated with SCC-7 cells which were pre-incubated with HA. c) The changes in fluorescent signals associated with endocytosis detected using flow cytometry with d) the statistical graph of mean fluorescence intensity, and e) the dynamic process observed using CLSM. f) The cytotoxic effects of hMnL and MnL treatments were evaluated. g) SCC-7 cells with various treatments observed by live/dead staining and imaged with CLSM. The apoptosis and necrosis of cells further analyzed by flow cytometry. h) the statistical graph and i) the representative flow images of the percent of early apoptosis, later apoptosis, necrosis and live cells. l) The changes in ROS levels within SCC-7 cells under different treatments detected using the DCFH-DA fluorescent probe, and j) fluorescence intensity variations analyzed by flow cytometry with k) the statistical analysis of mean fluorescence intensity. Data are expressed as mean ± SD (n = 3). ∗∗P < 0.01; ∗∗∗P < 0.001.

The nanoparticles designed in this study were surface-modified with HA, which could bind to the overexpressed CD44 receptors on SCC-7 tumor cells, providing sufficient binding sites for targeted delivery of the nanoparticle-based drug. Flow cytometric analysis of CD44 expression on HNSCC revealed that all SCC-7 cells expressed CD44 (Fig. 3a), as well as exhibiting relative high mean fluoresce intensity (Fig. 3b). To validate the role of HA in modulating the nanoparticle internalization efficiency, SCC-7 cells were pre-incubated with HA for 12 h, followed by the addition of fluorescently labeled hMnL. The internalization efficiency was then assessed by flow cytometry and CLSM. The results in Fig. 3c&d demonstrated that SCC-7 cells pre-treated with HA exhibited less uptake compared with without HA pretreatment. Besides, Fig. 3e showed that stronger fluorescence signals were observed in the cells without HA preincubation, indicating higher internalization efficiency, consistent findings with flow cytometric analysis. These results suggested that HA could mediate the internalization of nanoparticles by tumor cells. Furthermore, the cytotoxic effects induced by hMnL were examined in more detail. As shown in Fig. 3f, hMnL exhibited enhanced cytotoxicity in a dose-dependent manner. The surface modification with HA improved the nanoparticle targeting, thereby increasing the number of nanoparticles entering the cells and exerting stronger cytotoxic effects. Further live-dead staining confirmed these results, revealing an increased number of apoptotic cells (red) and a reduction in live cells (green) following hMnL treatment (Fig. 3g). Subsequently, flow cytometry was employed to assess cell apoptosis and necrosis. Compared to other groups, cells treated with hMnL showed a significantly higher level of apoptosis and necrosis, with approximately 80 % of the cells undergoing these processes (Fig. 3i). As can be observed from the statistical graph, tumor cells were induced to undergo extensive cell death, predominantly in the late apoptotic stage (Fig. 3h). Next, the impact of the internalized hMnL on cellular functions was explored. LAP was investigated for its ability to induce ROS production within cells, thereby promoting apoptosis. The effect of the nanoparticle formulations on ROS generation was further evaluated. The results revealed that LAP significantly elevated ROS levels (Fig. 3j&k). When LAP was encapsulated within hMn, the ROS generation was further enhanced, indicating that nanoparticle encapsulation and the inclusion of Mn significantly potentiated the cytotoxic effects on the cells. Images observed by CLSM also revealed that cells treated with hMnL exhibited stronger green fluorescence (Fig. 3l), in line with the results measured by flow cytometry.

### 3.3 Mitochondrial damage in tumor cells and ICD induced by hMnL

**Fig. 4:**
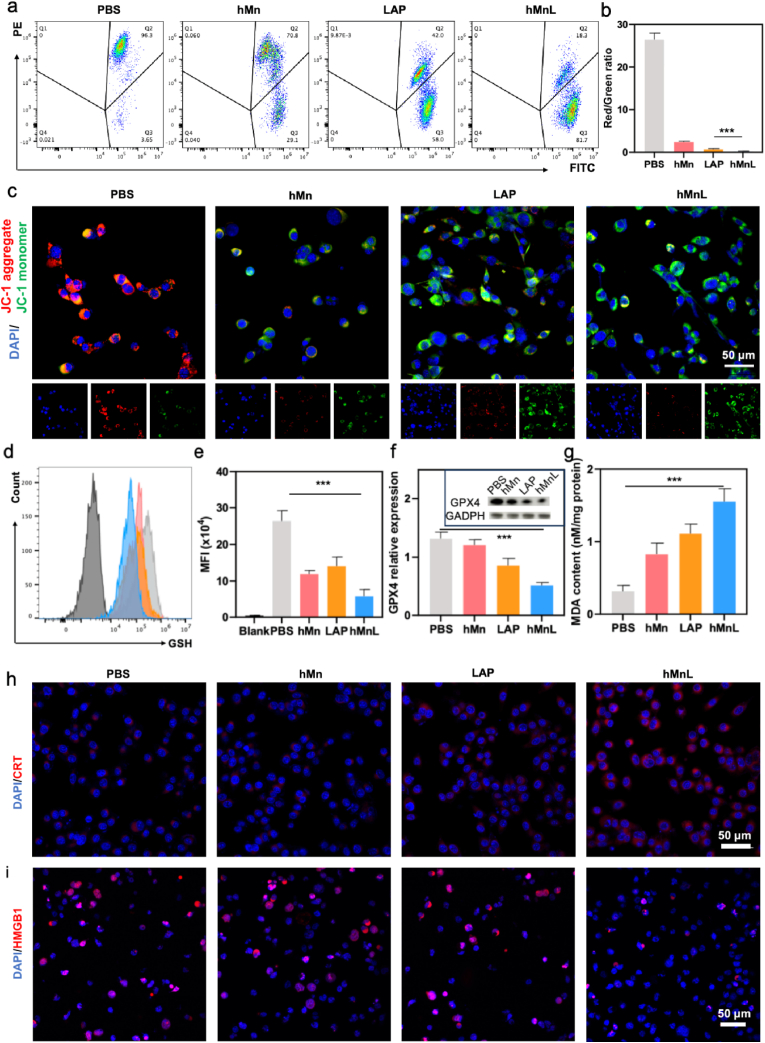
a) The changes in mitochondrial membrane potential of the cells measured using flow cytometry. b) The statistical graph of the ratio of red to green fluorescence intensity. Cells labeled with JC-1 dye after different induction conditions. c) SCC-7 cells with various treatments, stained with JC-1, and the changes in mitochondrial membrane potential observed using CLSM. Following various treatments, GSH levels in tumor cells measured. d) Quantification was performed using flow cytometry, with e) corresponding MFI values determined. f) The protein expression levels of glutathione peroxidase 4 (GPX4) in the treated tumor cells assessed by WB and subjected to quantitative analysis. g) MDA levels, indicative of lipid peroxidation, detected in cancer cells after various treatments. The expression of h) CRT and i) HMGB1 on SCC-7 cells after various treatments. Data are expressed as mean ± SD (n = 3). ∗∗∗P < 0.001.

The mechanism by which hMnL induced apoptosis in tumor cells was subsequently analyzed. Following various treatments, JC-1 staining was performed. Flow cytometric analysis revealed a significant decrease in the red/green fluorescence ratio of JC-1 after various treatments (Fig. 4a). Importantly, cells treated with hMnL exhibited the minimum red/green fluorescence ratio, indicating a substantial reduction in mitochondrial membrane potential (Fig. 4b). Additionally, after various treatments, the red fluorescence signal of JC-1 within the cells was markedly diminished, while the green fluorescence signal enhanced, observed by CLSM (Fig. 4c). Furthermore, the mitochondrial morphology transitioned from its usual tubular structure to a fragmented, granular form, further corroborating the loss of mitochondrial membrane potential. These findings collectively suggested that hMnL could induce mitochondrial damage in tumor cells, thereby triggering cell death. The altered mitochondrial membrane potential subsequently suggested potential induction of ferroptosis. Consequently, investigations were initiated to explore this association. As illustrated in Fig. 4d, flow cytometric analysis revealed a significant decrease in GSH expression after various treatments, and hMnL treated cells exhibited the most pronounced GSH depletion (Fig. 4e). Further validation *via* WB confirmed downregulation of GPX4, a key ferroptosis marker protein, in hMnL-treated cells (Fig. 4f). Malondialdehyde (MDA), a downstream biomarker of lipid peroxidation, was quantitatively assessed. Fig. 4g demonstrated a marked increase in intracellular MDA following hMnL treatment, providing additional evidence for ferroptosis activation. Collectively, these results indicated that hMnL triggered ferroptosis in cancer cells. Beyond ferroptosis, observation by CLSM of calreticulin (CRT) staining in variously treated cells revealed that cells exposed to hMnL exhibited the most intense CRT fluorescence signal (Fig. 4h). Images observed by CLSM revealed a significant decrease in HMGB1 fluorescence intensity within tumor cells following hMnL treatment, indicating its extracellular release (Fig. 4i). Further quantitative analysis showed that the concentration of ATP in the supernatant of the hMnL treated cells was the highest (Fig. S8). This result suggested that the hMnL could induce ICD in tumors.

## 4 BMDM and BMDC activation

**Fig. 5:**
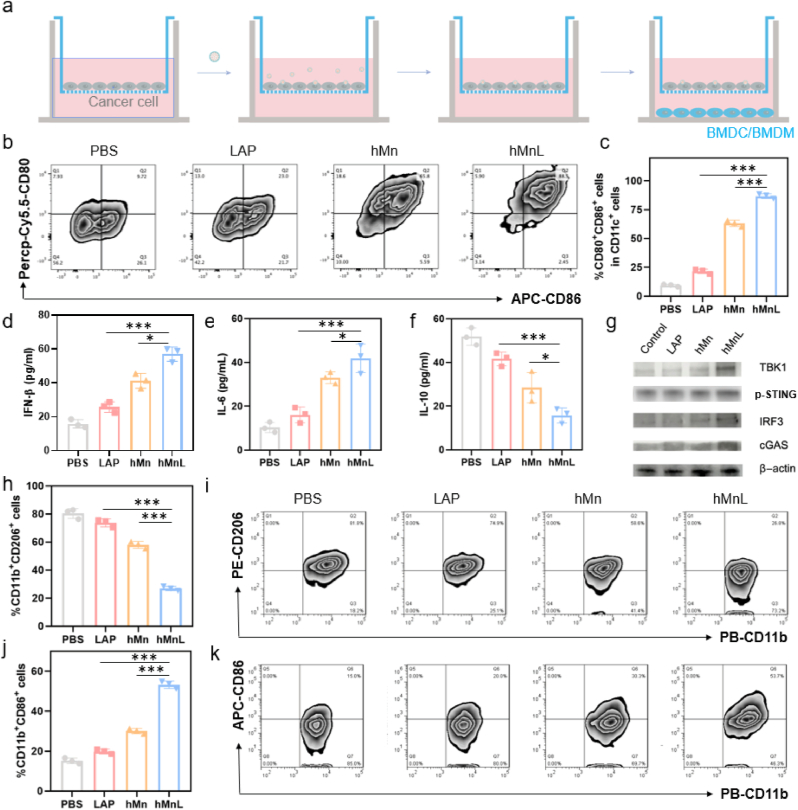
a) *In vitro* experiments conducted to evaluate the effects of activating BMDM and BMDC. The activation effects assessed through transwell assays. b) Representative flow cytometry plots displaying the expression levels of CD80 and CD86 in BMDC under different treatment conditions, accompanied by c) corresponding quantitative statistical graphs. Cytokine levels in the supernatant of BMDC culture medium measured, focusing on d) IFN-β, e) IL-6, and f) IL-10. The polarization of macrophages was analyzed, and the expression levels of CD206 were quantified, with related flow cytometry plots provided. g) BMDM stimulated with PBS, LAP, hMn and hMnL for 24 h, respectively, the expression changes of related proteins were detected using western blotting. h) The expression levels of CD206 also quantitatively analyzed, and i) representative flow cytometry plots included. j) The expression levels of CD86 quantitatively analyzed, and k) representative flow cytometry plots included. Data are expressed as mean ± SD (n = 3). ∗P < 0.05; ∗∗∗P < 0.001.

hMnL could induce ICD in tumor cells, thereby further activating immune cells. Additionally, manganese within the hMnL released after inducing ICD of cancer cells, which, in conjunction with cells debris, promoting an immune response. To assess this process, an *in vitro* model was established, as illustrated: tumor cells were positioned in the upper chamber, and after various treatments, immune cells in the lower chamber, including BMDM and BMDC, were activated *via* the Transwell system (Fig. 5a). As shown in Fig. 5b&c, treatments with LAP and hMn led to an increased expression of CD80 and CD86, while the hMnL treatment exhibited the most robust expression of these markers. This indicated that hMnL treatment induced BMDC maturation and, in synergy with Mn^2+^, further activated BMDC. The enhanced effect was attributed to the activation of the STING pathway by Mn^2+^. Moreover, the expression levels of IFN-β (Fig. 5d), IL-6 (Fig. 5e), and IL-10 (Fig. 5f) in the collected cell supernatants were significantly elevated, suggesting that this treatment promoted an inflammatory response conducive to DC activation. Additionally, the activation of the STING signaling pathway in BMDM was evidenced by the upregulated expression levels of key pathway proteins, including cGAS, p-STING, TBK1, and IRF3 (Fig. S9). Specifically, cells in hMnL group exhibited the most pronounced upregulation of these proteins (Fig. 5g). Similarly, the treatments also facilitated the polarization of M1-type macrophages, evidenced by a decrease in CD206 expression (Fig. 5h&i) and an increase in CD86 expression (Fig. 5j&k). Notably, the hMnL group significantly upregulated CD86 expression, an effect also mediated by Mn activation of the STING pathway in APCs. These results collectively demonstrated that hMnL induced ICD in tumor cells activating immune cells, thereby contributing to anti-tumor efficacy. The causal relationship between cell death and immune activation was investigated. As shown in Fig. S6, hMnL treatment induced the release of cytoplasmic dsDNA, a phenomenon that was significantly inhibited by a ferroptosis inhibitor (Ferrostatin-1), confirming that the treatment primarily triggered ferroptosis. Subsequently, the results in Fig. S6 also demonstrate that hMnL treatment upregulated the expression of phosphorylated TBK1 (*p*-TBK1), indicating activation of the STING pathway. Notably, this activation could likewise be reversed by Ferrostatin-1. Collectively, these data prove that hMnL-induced ferroptosis served as a key upstream event driving the activation of STING pathway.

### 4.1 RNA-seq analysis of hMnL

**Fig. 6:**
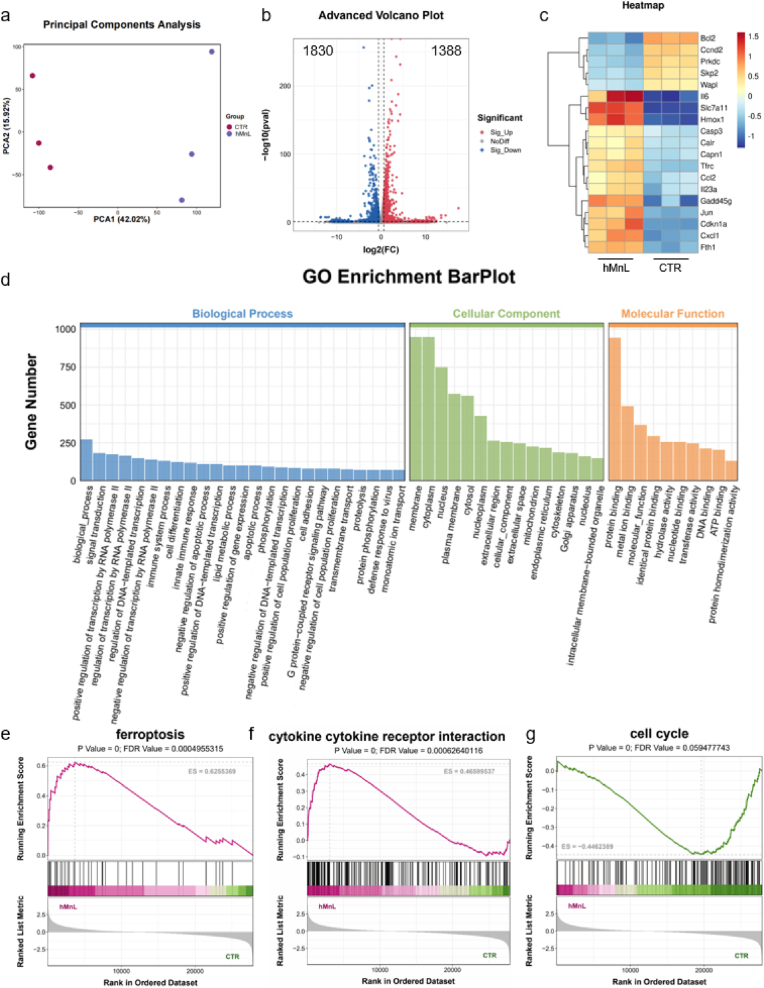
a) PCA based on bulk RNA-seq results of the PBS (CTR) and hMnL. b) Volcano map of the transcriptomic profiles. c) Heat map of mRNAs related to treatment progression. d. Gene Ontology analysis of hMnL treated group as compared with the CTR group. GSEA of e) ferroptosis apoptosis f) cytokine cytokine receptor interaction and g) cell cycle.

Further mechanistic investigation was conducted *via* RNA sequencing (RNA-seq) to evaluate the therapeutic mechanisms of hMnL in cells. Principal component analysis (PCA) revealed distinct clustering between the hMnL treated group and the control group (Fig. 6a). Bioinformatic analysis was subsequently performed to compare differential gene expression (Fig. 6b). The results identified 1830 downregulated and 1388 upregulated differentially expressed genes in hMnL treated cells. As shown in Fig. 6c, the mRNA heatmap illustrates transcriptomic alterations following hMnL treatment. Key pathways implicated in the therapeutic effects of hMnL were then analyzed through enrichment analysis. Gene Ontology (GO) enrichment analyses indicated significant enrichment of apoptosis-related processes, immune responses, mitochondrial function, among others (Fig. 6d). These findings suggested that hMnL treatment exerted anti-cancer effects partially through these mechanisms. Furthermore, gene set enrichment analysis (GSEA) was applied to the RNA-seq data to better elucidate hMnL induced transcriptomic changes. As anticipated, three significantly enriched pathways were identified, including ferroptosis (Fig. 6e), cytokine cytokine receptor interaction (Fig. 6f), and cell cycle (Fig. 6g). Corresponding pathway-related genes were also represented in the heatmap, such as *Fth1* and *Hmox1* (ferroptosis), *Il6* and *Ccl2* (cytokine cytokine receptor interaction), as well as *Atm* and *Skp2* (cell cycle). In conclusion, these results implied that hMnL may effectively kill cancer cells by inducing ferroptosis and immunogenic cell death, while the cytokines secreted by cells may also promote the infiltration of immune cells, such as T cells.

### 4.2 *In vivo* active delivery in a solid tumor model

**Fig. 7:**
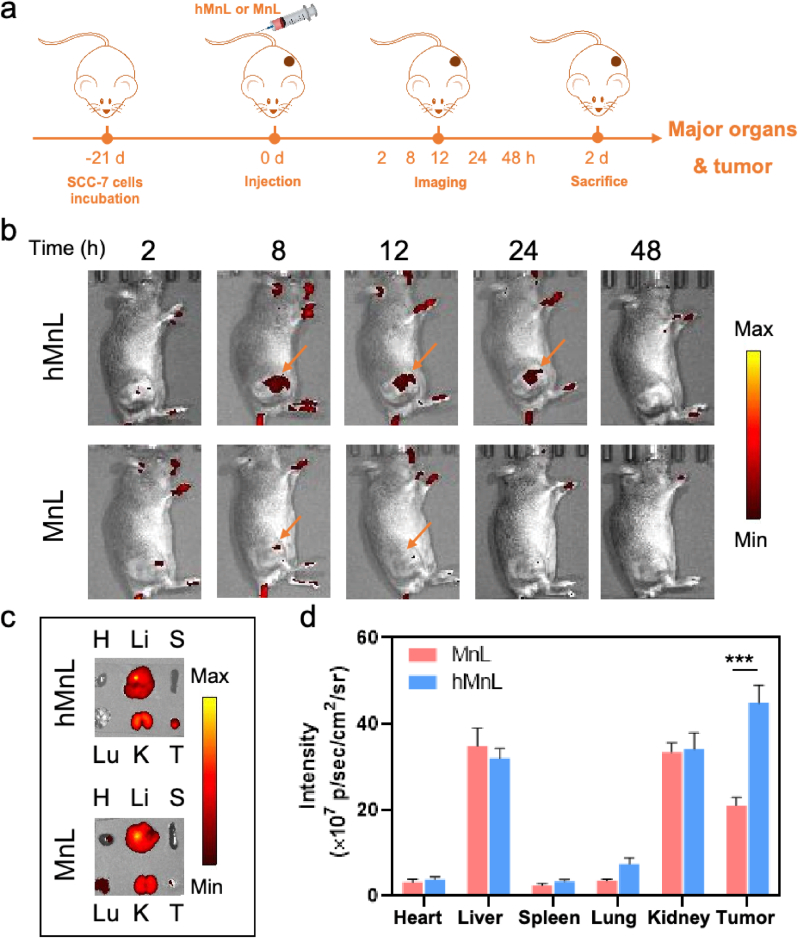
hMnL could efficiently accumulated in SCC-7 tumor areas. a) Schematic of tumor-bearing mouse model construction and fluorescence labeled hMnL, MnL were administered to the mice to assess the targeting efficacy. b) *In vivo* fluorescence biodistribution of Cy7 loaded hMnL or MnL in SCC-7 tumor-bearing mice and c) *ex vivo* Cy7 fluorescent images of tumor and major organs (heart, liver, spleen, lung, kidney) 48 h post-injection. d) ROI analysis of major organs and tumors. Data are expressed as mean ± SD (n = 3). ∗∗∗P < 0.001.

The ability of hMnL to specifically target and efficiently circulate to tumor tissues was crucial for the subsequent anti-tumor effects. To evaluate the ability, a tumor-bearing mouse model was constructed and fluorescence labeled hMnL and MnL were administered to the mice, assessing the targeting efficacy of the nanoparticles at the tumor site, as depicted in the schematic (Fig. 7a). As shown in Fig. 7b, hMnL exhibited significantly superior accumulation at the tumor site compared to unmodified HA nanoparticles (MnL). Notably, during the initial hours post-treatment, the hMnL displayed stronger fluorescence at the tumor site. In contrast, the unmodified HA nanoparticles showed weaker accumulation, suggesting enhanced tumor targeting due to HA modification. However, over time, the fluorescence intensity gradually decreased. To further investigate the distribution among various organs, the mice were euthanized to harvest major organs and tumors for imaging. The results demonstrated that, as shown in Fig. 7c, the fluorescence intensity in the tumors of hMnL group mice was significantly higher than that of the MnL group, and quantitative fluorescence analysis confirmed this trend (Fig. 7d). In conclusion, under the mediation of HA, hMnL was able to selectively accumulate in tumor tissues, thus providing a foundation for subsequent anti-tumor therapy.

### 4.3 Chemo*-*immunotherapeutic effects in HNSCC tumor model

**Fig. 8:**
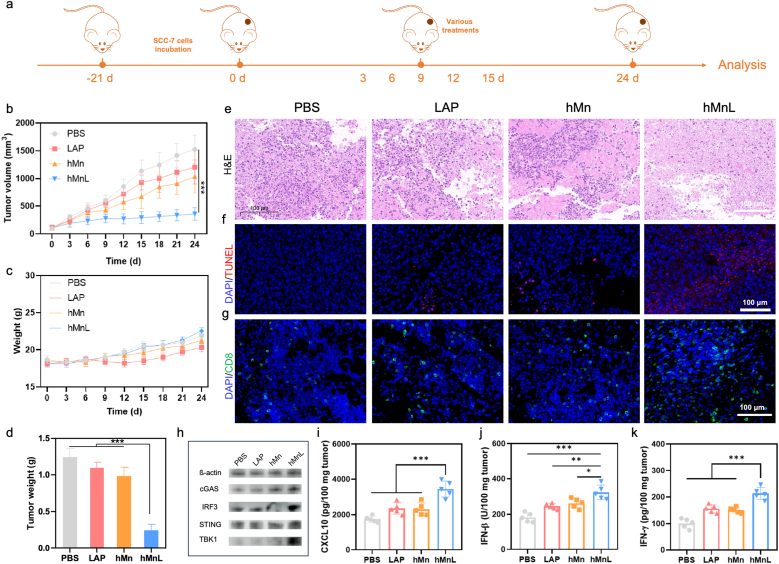
a) Schematic of tumor-bearing mouse model construction, various treatments, and result analysis. b) Monitoring of tumor growth curve. c) Body weight measurements of mice during the treatment period. d) Statistical analysis of tumor mass in mice. e) H&E, f) TUNEL and g) CD8^+^ T cell staining of tumor tissue sections at the end of treatment. h) Expression levels of cGAS-STING pathway proteins in tumor tissues of mice after various treatments. i) The secretion of cGAS-STING pathway related cytokines, including i) CXCL10, j) IFN-β and proinflammatory cytokines k) IFN-γ. Data are expressed as mean ± SD (n = 5). ∗P < 0.05, ∗∗P < 0.01, ∗∗∗P < 0.001.

After evaluating the efficient accumulation of hMnL in tumor tissues, *in vivo* antitumor efficacy was further investigated. As illustrated in the timeline (Fig. 8a), tumor-bearing mouse models were established. After different treatments were administered to the mice, various parameters were regularly monitored. At the conclusion of the experiment, the mice were euthanized, and tissues were extracted for the preparation of single-cell suspensions to assess the expression levels of immune cells. The results indicated that, in the group treated with PBS, the tumor volume increased rapidly over the 24-day period and was the largest among all groups (Fig. 8b). Following treatment with the chemotherapeutic agent LAP, the tumor volume of the mice was significantly reduced, suggesting that the drug exhibited some inhibitory effects on tumor growth. Similarly, the group treated with hMn also demonstrated a reduction in tumor volume. Notably, hMnL group showed a marked inhibition of tumor growth, with results significantly superior to those of the other groups. This finding suggested that hMnL, once targeted and enriched within the tumor tissue, exerted a tumoricidal effect while also activating immune-mediated tumor cell destruction. During the treatment, the body weight of the mice was monitored and no significant changes were observed in hMnL treated mice (Fig. 8c). Upon completion of the treatments, the mice were euthanized and the tumors were excised from the mice to weigh. As depicted in Fig. 8d, the tumors in the mice of hMnL group exhibited the lowest mass. Further investigation was required to elucidate the detailed mechanisms underlying the effects of hMnL.

To further elucidate the superior efficacy of hMnL, tumor sections were analyzed. H&E staining revealed significant histological alterations in the tumor tissues of treated mice. Compared to PBS group, the tumor tissues in the hMnL-treated group exhibited pronounced nuclear condensation and fragmentation, indicative of apoptosis or necrosis (Fig. 8e). Additionally, the cellular arrangement within the tumor tissue was more disorganized, with irregular cell morphology and evident structural disruption of the extracellular matrix. These findings suggested that the hMnL treatment substantially affected the tumor cell morphology and likely induced apoptotic or necrotic cell death, thereby providing further evidence for the antitumor activity of the treatment. Subsequent immunofluorescence analysis revealed that the tumor tissues of mice with various treatments exhibited the strongest TUNEL fluorescence, indicating effective induction of DNA damage in the tumor tissue (Fig. 8f). TUNEL staining results demonstrated a significant increase in the number of apoptotic cells across various treatments (hMn, LAP, hMnL). Compared to PBS group, hMn, LAP, and hMnL-treated groups displayed more intense red fluorescence signals, suggesting prominent DNA fragmentation and a higher degree of apoptosis. Notably, in the hMnL treated tumor tissues of mice, the distribution of stained cells was more widespread, further suggesting that the drug effectively induced apoptosis in tumor cells.

Inspired by the extracellular activation of the STING pathway, *in vivo* STING pathway activation in tumor-bearing mouse model was evaluated. Mn^2+^ released from hMnL stimulated cGAS, promoting STING pathway activation and DC maturation. The expression of cGAS-STING pathway related proteins and cytokine secretion in tumor tissues were determined. As shown in Fig. 8h, compared with PBS group, hMnL group exhibited significantly elevated expression levels of STING, cGAS, TBK1, and IRF3. Increased STING fluorescence signals were also observed in tumor tissue sections after hMnL treatment, indicating that the tumor microenvironment-driven release of Mn^2+^ could enhance STING-mediated type I interferon production (Fig. S2). Meanwhile, secretion of cGAS-STING related cytokines CXCL10 (Fig. 8i) and IFN-β (Fig. 8j) and pro-inflammatory cytokines including IFN-γ (Fig. 8k), IL-12 (Fig. S3a), IL-6 (Fig. S3b) and TNF-α (Fig. S3c), was significantly increased in hMnL group compared with other groups, supporting cGAS-STING pathway activation and the potential to convert “cold” tumors into more responsive “hot” tumors. Therefore, hMnL induced activation of the cGAS-STING pathway, associated with IFN-β secretion, may contribute to DC maturation, thereby inducing subsequent T-cell activation.

### 4.4 Analysis of immune mechanism

**Fig. 9:**
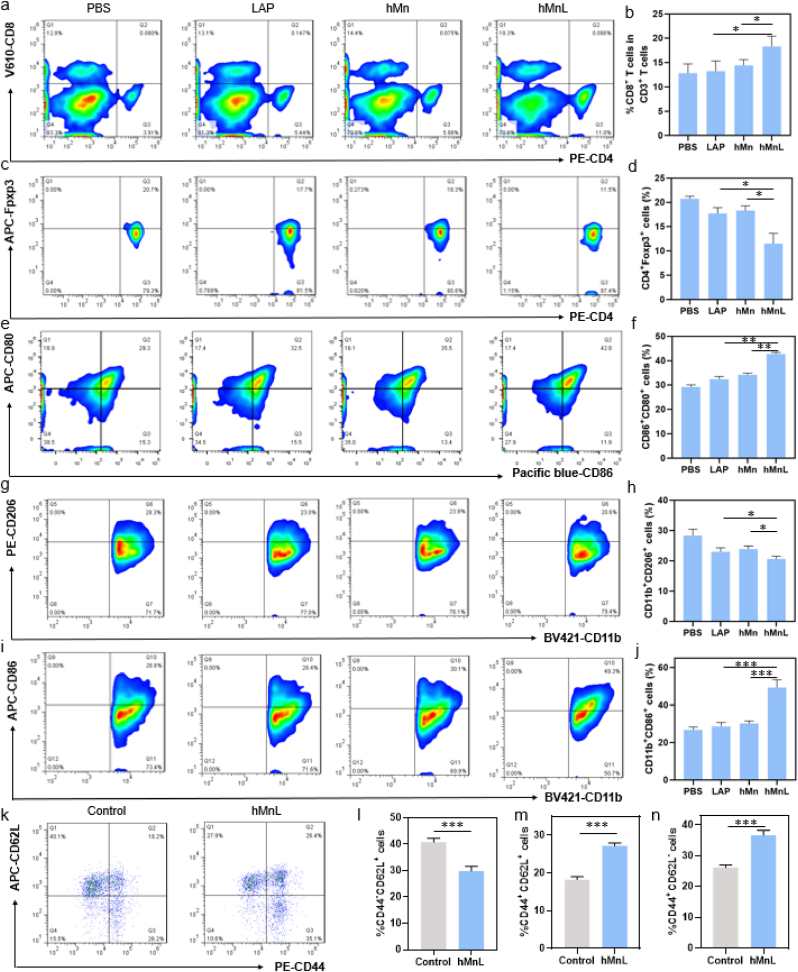
a) The infiltration of CD8^+^ T cells in tumor tissue analyzed using flow cytometry, along with b) corresponding statistical graphs. c) The number of Treg cells in the tumor and d) the associated statistical data. The phenotype of DC cells in the lymph node, including e) the representative flow cytometry plots of the expression levels of CD80 and CD86 and f) statistical graphs provided. The macrophage phenotype in tumor tissue was analyzed, including g) the representative flow cytometry plots of the expression of M2-type macrophages and h) related statistical data, as well as i) the expression of M1-type macrophages and j) statistical graphs. hMnL therapy enhanced lymphocyte activation and proliferation in spleen. k) Representative flow cytometry plots showing the percentages of CD44 and CD62L T cells in the spleen. Cumulative results of l) CD44^−^CD62L^+^ T cells, m) CD44^+^CD62L^+^ T cells and n) CD44^+^CD62L^−^ T in the spleen. Data are expressed as mean ± SD (n = 3). ∗P < 0.05; ∗∗P < 0.01; ∗∗∗P < 0.001.

To further investigate the anti-tumor immune effects, the infiltration of CD8^+^ T cells in tumor tissues from mice was analyzed through immunofluorescence staining. As shown in Fig. 8g, a significant increase in CD8^+^ T cell infiltration was observed in tumor tissues across various treatments. Compared to PBS group, hMnL exhibited a marked elevation in the number of CD8^+^ T cells within the tumor tissue, indicating that the treatment effectively induced the recruitment of cytotoxic T cells and enhanced their infiltration into the TME. Flow cytometry analysis of infiltrating CD8^+^ T cells further revealed that hMnL treated tumors exhibited the highest CD8^+^ T cell infiltration (Fig. 9a), approximately 1.5-fold than that of PBS group (Fig. 9b), consistent with the trends observed by immunofluorescence staining. The improvement of the immunosuppressive microenvironment facilitated alterations in other immune cell populations within the tumor. Further analysis of T cell subsets showed a notable reduction in Treg cells following hMnL treatment (Fig. 9c), with the number being approximately half that of PBS group, suggesting that the immunosuppressive TME was significantly ameliorated (Fig. 9d). Next, the mechanisms underlying immune activation were explored by analyzing the phenotypes of infiltrating DCs and macrophages in the tumor tissue, as nanoparticles were known to activate the STING pathway. As depicted in Fig. 9e, various treatments led to substantial changes in the phenotype of DCs within the lymph node. Compared to PBS group, hMn group exhibited a significant increase in the expression of CD80^+^CD86^+^ on DCs, likely due to Mn^2+^ accumulation in the tumor tissue, which activated the STING pathway and subsequently enhanced DC activation (Fig. 9f). Importantly, hMnL induced the strongest activation of DCs, suggesting the effectively improved tumor suppressive microenvironment, thereby facilitating immune responses. Additionally, compared to other groups, hMnL treatment exhibited the most pronounced macrophage polarization (Fig. 9g and h), with macrophages in the tumor tissue skewing towards the M1 phenotype (Fig. 9i and j), further supporting the enhanced anti-tumor immune response.

Furthermore, hMnL may exhibit long-lasting immunoprotective effects, as they had the potential to induce enduring immune memory. The establishment of immune memory originated from memory T cells. In this study, to evaluate the long-term immune memory induced by hMnL treatment, mice were euthanized on day 14 after the final administration for splenic harvest and flow cytometric analysis. A reduction in naive T cells (CD44^−^CD62L^+^) in mice following hMnL treatment was observed (Fig. 9k&l), concomitant with a significant increase in central memory T cells (TCM, CD44^+^CD62L^+^) (Fig. 9m) compared to control mice. Additionally, an upward trend was noted in effector memory T cells (TEM, CD44^+^CD62L^−^) (Fig. 9n). These findings suggested that the immune system was activated, with T cells transitioning to effector or memory states, potentially indicating therapeutic efficacy and the formation of long-term immune memory.

In summary, treatment with hMnL significantly enhanced the anti-tumor immune response by promoting the infiltration of CD8^+^ T cells into the TME. Flow cytometry analysis confirmed that hMnL treatment induced the highest level of CD8^+^ T cell infiltration, approximately twice that of the control group. This was accompanied by a reduction in Treg cells, indicating an improvement in the immunosuppressive TME. Furthermore, hMnL treatment activated DCs and macrophages, with the strongest DC activation observed in the hMnL group, likely due to Mn^2+^ activation of the STING pathway. Additionally, hMnL treatment promoted macrophage polarization towards the M1 phenotype, further enhancing the immune response. These findings suggested that hMnL not only targets tumor cells directly, but also modulated the immune microenvironment to support anti-tumor immunity.

**Fig. 10:**
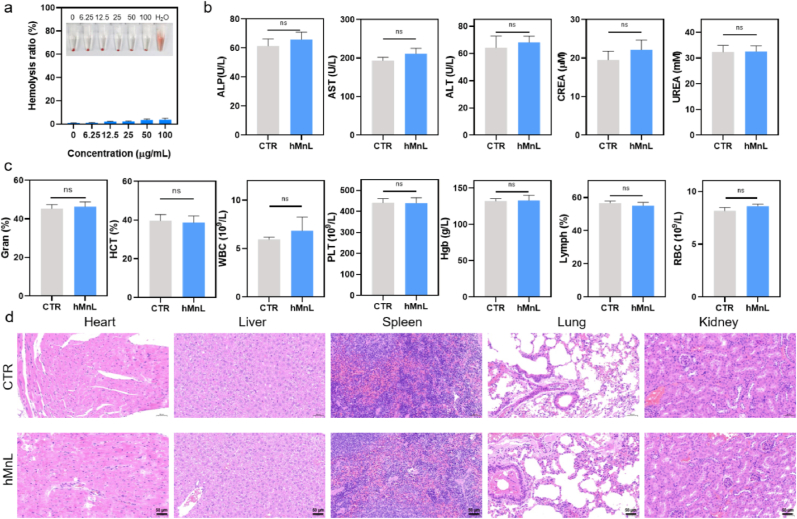
Biosafety studies of hMnL. a) Quantitative analysis after hMnL treatment at different concentrations (0–100 μg/mL) and corresponding images. b) Liver and kidney function evaluation *via* analysis of blood serum. c) Hematological examination. d) H&E staining of major organs of healthy mice including heart, liver, spleen, lung and kidney, after hMnL treatment for four weeks. Data are expressed as mean ± SD (n = 3). n.s., not significant. (ALP: alkaline phosphatase, AST: aspartate aminotransferase, ALT: alanine transaminase, CREA: creatinine, UREA: urea, Gran%: c, HCT%: hematocrit ratio, WBC: white blood cells, PLT: platelet, Hgb: hemoglobin, Lymph%: lymphocyte ratio, RBC: red blood cells).

The long-term biosafety of hMnL was evaluated, beginning with an *in vitro* hemolysis assay to assess its hemocompatibility. As shown in Fig. 10a, hMnL at various concentrations (0–100 μg/mL) did not induce significant hemolysis, which was further reflected by the absence of notable changes in supernatant absorbance. This finding provided a foundation for the safety of *in vivo* hMnL administration. Additionally, *in vivo* studies were carried out to evaluate the histocompatibility of hMnL. ICP-MS analysis (at 18 h post-injection) revealed that manganese was predominantly distributed in the liver and kidney (Fig. S7), which is consistent with the normal metabolic pathway as reported. Monitoring of mice body weight revealed no evidence of toxicity attributable to hMnL administration (Fig. S4). The analysis of biomarkers in mice peripheral blood showed that hMnL did not cause alterations in biochemical or hematological parameters, suggesting negligible inflammatory responses (Fig. 10b and c). H&E staining was performed to conduct histopathological analysis on major organs, including heart, liver, spleen, lung, and kidney. The results showed no significant differences in the tissue structures of the major organs after hMnL treatment, with no observed pathological changes such as inflammation, necrosis, or fibrosis (Fig. 10d). Collectively, the *in vitro* hemocompatibility assessment and *in vivo* histocompatibility evaluation demonstrated that hMnL possessed favorable long-term biosafety and showed promising potential for clinical translation.

This therapeutic system demonstrated excellent efficacy in HNSCC, highlighting the importance of expanding its broad-spectrum applicability. Consequently, hMnL was applied to another cancer model characterized by peritoneal dissemination-ovarian cancer. As shown in Fig. S5, tumor-bearing mice injected solely with PBS exhibited sustained and rapid tumor growth. When treated with LAP and hMn, tumor progression in mice was significantly suppressed. However, tumor volume remained substantial by the end of the treatment cycle. In contrast, administration of hMnL resulted in continuous inhibition of tumor growth throughout the study. Ultimately, tumors in these mice were maintained at significantly reduced volumes. This outcome demonstrated the pronounced ability of hMnL to inhibit tumor growth and exhibited the broad-spectrum anti-tumor effects.

## 5 Conclusions

In this study, an effective therapeutic platform for HNSCC treatment was developed with promising potential. By leveraging the combined advantages of MnO_2_-based nanoparticles, HA surface modification, and chemotherapeutic drug loading, hMnL demonstrated enhanced tumor targeting, effective drug release in response to the TME, and a potent antitumor effect through immune modulation. It has been demonstrated that hMnL effectively accumulated in tumor tissues, leading to significant tumor growth inhibition. Importantly, treatment with hMnL significantly improved the TME by promoting the infiltration of cytotoxic CD8^+^ T cells, while also reducing the population of immunosuppressive Treg cells. The activation of the STING pathway in antigen-presenting cells further bolstered the anti-tumor immune response, with enhanced macrophage polarization towards the M1 phenotype. Intriguingly, hMnL exhibited desired immunoprotective effect. These findings highlight the dual mechanism of action of hMnL, both as a direct chemotherapeutic agent and as an immunomodulator, positioning it as a promising candidate for combination therapies aimed at improving cancer treatment efficacy. Importantly, hMnL exhibits favorable long-term biosafety and holds considerable translational potential. Overall, this study provided a novel strategy for targeted, effective cancer therapy that not only addressed the primary tumor, but also reprogrammed the TME to facilitate sustained anti-tumor immunity.

## CRediT authorship contribution statement

**Wa Li:** Project administration, Writing – original draft. **Zihui Tang:** Investigation, Writing – original draft. **Jiyang Xue:** Supervision, Writing – review & editing.

## Declaration of competing interest

The authors declare that they have no known competing financial interests or personal relationships that could have appeared to influence the work reported in this paper.

## Data Availability

Data will be made available on request.
