# Supplementary material for: Synergistic STING activation and oxidative cascades-induced ferroptosis drive tumor microenvironment remodeling by engineered manganese nanoreactors
Source: Redox Biol. 2025 Dec 15;89:103977. doi: 10.1016/j.redox.2025.103977 (PMC12808843; doi:10.1016/j.redox.2025.103977)
Supplement: Multimedia component 1 [file mmc1.docx]

**Synergistic STING Activation and Oxidative Cascades-Induced Ferroptosis Drive Tumor Microenvironment Remodeling by Engineered Manganese Nanoreactors**


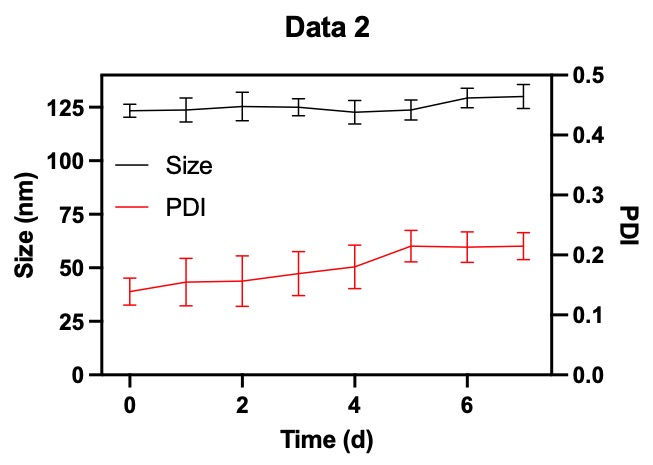


**Figure S1** Size and PDI of hMnL in PBS measured every 24 h


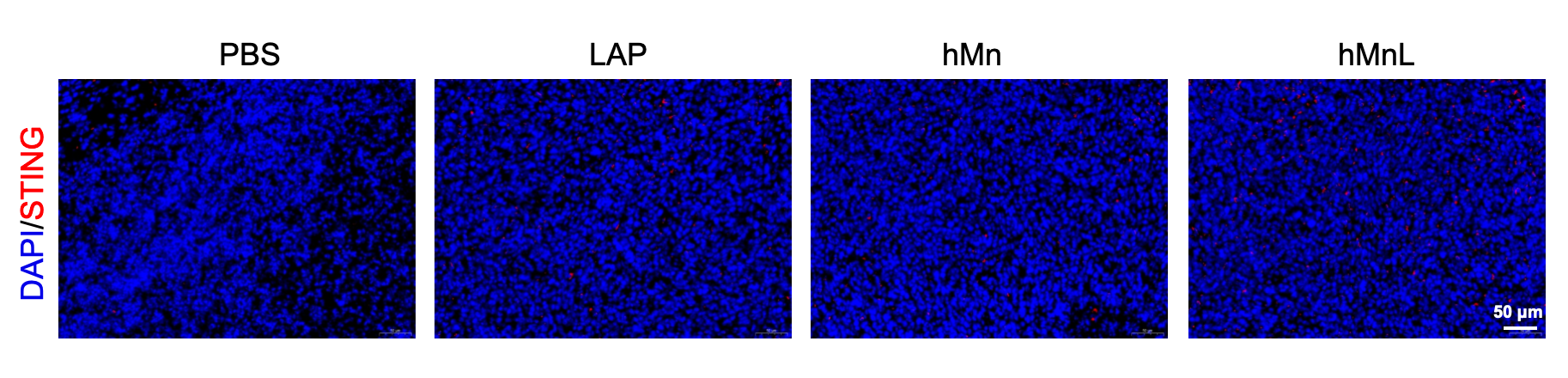


**Figure S2** STING protein expression in tumor tissue sections at the end of treatment.


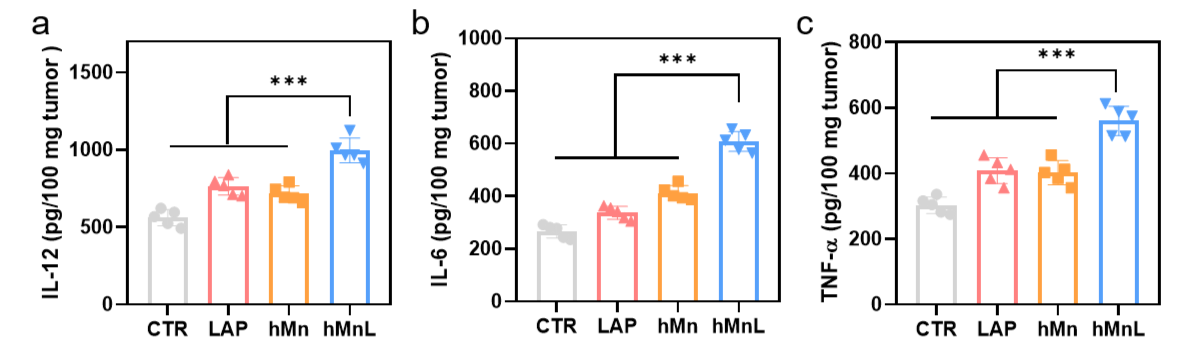


**Figure S3** The secretion of proinflammatory cytokines a) IL-12, b) IL-6 and c) TNF-α. Data are expressed as mean ± SD (n = 5). ***P < 0.001.


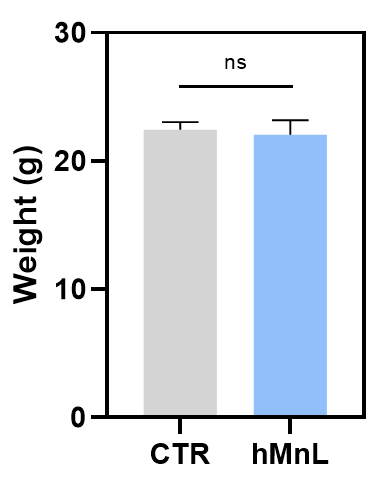


**Figure S4** Mice weight after hMnL treatment. Data are expressed as mean ± SD (n = 3). n.s., not significant.


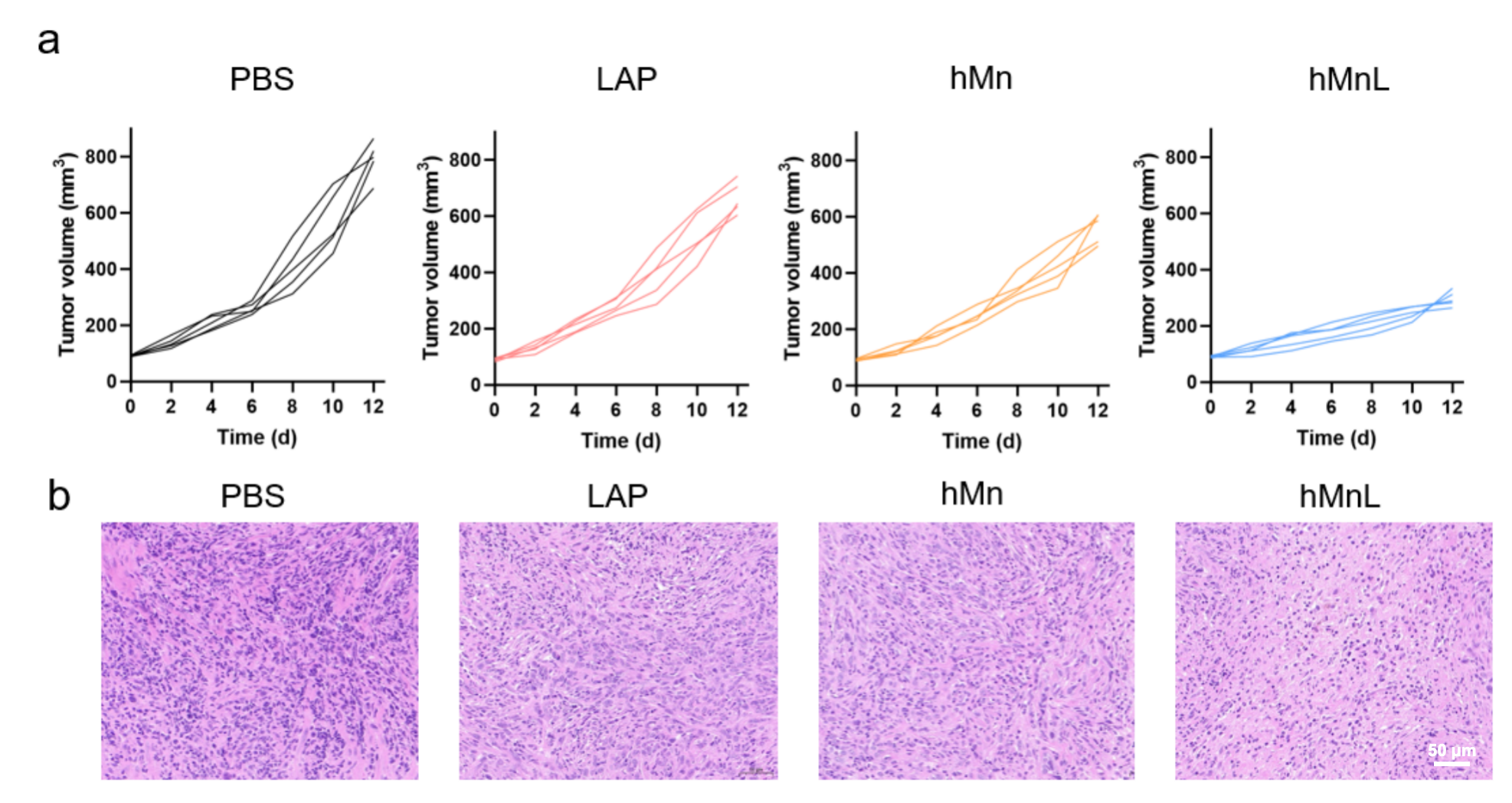


**Figure S5** a) Tumor-growth profiles of ovarian tumor-bearing mice receiving indicated treatments. b) H&E staining of tumor tissue.


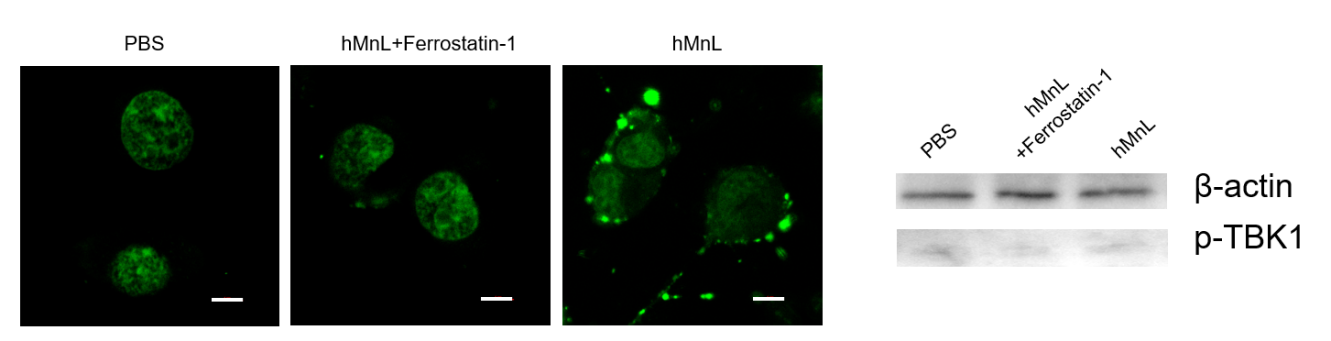


**Figure S6** dsDNA expression in cells following different treatments, and p-TBK1 expression in DC cells.


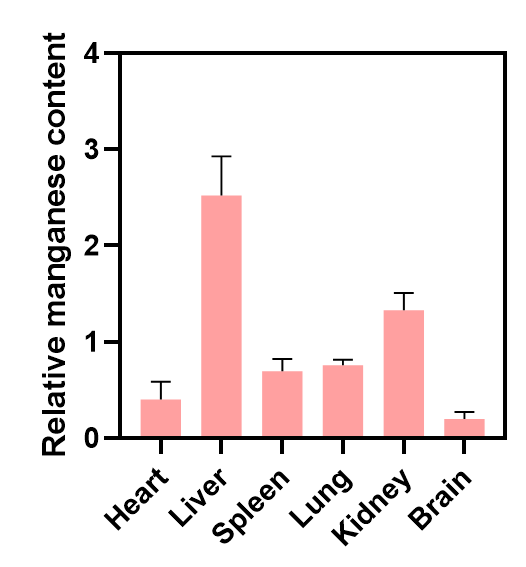


**Figure S7** Copper distribution across various tissues.


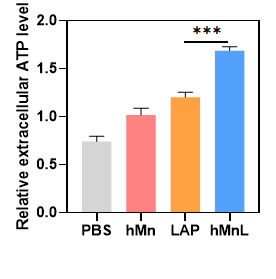


**Figure S8** ATP expression of cells after different treatments (n=3).


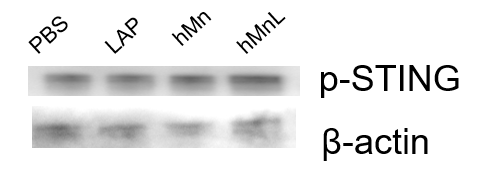


**Figure S9** The expression of p-STING.
